# Novel vs clinical organ preservation solutions: improved cardiac mitochondrial protection

**DOI:** 10.1186/s13019-017-0564-x

**Published:** 2017-01-26

**Authors:** Alice S. Ferng, David Schipper, Alana M. Connell, Katherine M. Marsh, Shannon Knapp, Zain Khalpey

**Affiliations:** 1 Division of Cardiothoracic Surgery, Department of Surgery, University of Arizona College of Medicine, Tucson, AZ USA; 2 Department of Physiological Sciences, University of Arizona College of Medicine, Tucson, AZ USA; 3 Department of Biomedical Engineering, University of Arizona College of Medicine, Tucson, AZ USA; 4 University of Arizona College of Medicine, Tucson, AZ USA; 5 University of Arizona College of Medicine, Statistics Consulting Lab, BIO5 Institute, Tucson, AZ USA; 6 Banner – University Medical Center, Tucson, AZ USA

**Keywords:** Organ preservation solution, Cardiac myoblasts, Mitochondria, Bioenergetics, Somah, Celsior, Perfadex

## Abstract

**Background:**

Heart transplantation remains the gold standard for end-stage heart failure, with current ex vivo organ storage times limited to 4 to 6 h before critical tissue damage occurs. Many preservation solutions exist in an attempt to limit both ischemic and reperfusion damage. In order to compare the effects of various storage solutions, mitochondrial function can be used to provide a sensitive analysis of cellular metabolic function.

**Methods:**

Experimental plates were seeded with cardiac myoblasts and kept in suspended animation for either 4 or 8 h at either 4^o^ or 21 °C, in Celsior®, Perfadex®, or Somah storage solutions. Cells were then reanimated for 1 h at 37 °C to simulate a reperfusion or clinical transplant scenario. Cellular bioenergetics were measured immediately thereafter to examine biochemical differences between preservation solutions and their effectiveness on preserving metabolic function.

**Results:**

The oxygen consumption rates of Somah solution were significantly higher than Celsior® and Perfadex® at 4 °C, with the exception of Perfadex® at 4^o^ for 4 h. This effect was sustained up to 8 h. At 21 °C, oxygen consumption rates of Somah solution are significantly higher than Celsior® and Perfadex® at basal conditions after 4 h, but this effect is not sustained after 8 h.

**Conclusions:**

The purpose of this experiment was to study the efficacy of various preservation solutions on a mitochondrial level. The significantly higher oxygen consumption rates of Somah at 4 °C suggests that Somah solution may have the ability to protect cellular mitochondrial integrity, improve transplanted organ function by reducing ischemic-reperfusion injury, and thereby improve transplant outcomes. Given that Somah offers benefits over Celsior® and Perfadex® at 4 °C, it should be a target in future organ preservation solution research.

**Electronic supplementary material:**

The online version of this article (doi:10.1186/s13019-017-0564-x) contains supplementary material, which is available to authorized users.

## Background

The gold standard for patients suffering from end-stage heart failure remains heart transplantation. With the number of patients requiring transplants growing and the available organs remaining constant, there is a need for improved donor organ and graft preservation methods to extend the life of donor organs [1]. Conserving organ viability outside of an organism can be accomplished by warm or cold storage, with or without perfusion. Currently, the standard approach in heart preservation is cold static storage, often supplemented by the use of preservation solutions [2]. These approaches have allowed for an ex vivo storage time of about 4–6 h before critical tissue damage occurs [3]. Preservation of organs for longer than 4–6 h results in increased ROS levels, ATP depletion, Na^+^/K^+^ ATPase alterations, mitochondrial disturbances, accumulation of xanthine oxidase, and dysregulation of Ca^2+^ homeostasis that will negatively effect cellular viability [2, 4]. Under normal physiological conditions, the heart utilizes ATP as energy for the Na^+^/K^+^ ATPase, both of which are required for sustained myocardial contractility. During ischemia, ATP levels decrease while intracellular H^+^ increases due to the shift from aerobic to anaerobic respiration via glycolysis and lactate production. ATPase pumps that maintain homeostasis become dysfunctional as the synthesis of ATP slows. Disruption of these ATPase pumps in the mitochondria can be measured experimentally through examining extracellular flux and mitochondrial respiration. In an attempt to avoid the negative effects of decreased ATPase pump efficacy, both the cold storage approach and utilization of storage solutions exists.

Cellular metabolic demand decreases as much as 12-fold under hypothermic conditions at 4 °C [5], but metabolic processes persist and will continue to result in cellular damage. Though warm preservation might result in injury from non-controlled warm ischemic periods [2], it is less researched and could be a more effective and sophisticated storage method [6]. Regardless of storage temperature during perfused or static storage, preservation solutions such as Celsior®, Perfadex®, and Somah storage solutions are commonly used to decrease ischemic reperfusion injury (IRI) from reactive oxygen species (ROS), prevent intra- and extracellular swelling, and minimize energy usage by lowering metabolic demand. The conservation of mitochondrial bioenergetic measurements such as these is important for improving organ transplant outcomes during static storage. During storage and upon reperfusion, IRI can be attenuated by using storage solutions such as University of Wisconsin (UW) solution [7]. However, UW contains high molecular weight compounds such as hydroxyethyl starch that resulted in a highly viscous solution that is linked to organ dysfunction. Newer alternatives including Celsior®, Perfadex®, and Somah have since been created to allow for reliable organ preservation up to 6 h by providing immunosuppressant and antioxidant properties that minimize IRI [4, 7]. There are numerous organ storage solutions, with many new solutions currently in development. Somah is a novel solution that acts to maintain membrane polarity by allowing higher levels of high-energy phosphates to be generated through the glycolytic pathway during preservation, and mitigates the consequences of IRI overall [8]. Somah was developed to meet the energy requirements of cardiomyocytes and coronary endothelium, in addition to priming the organ with substrates and metabolites during storage to facilitate resumption of biochemical, physiological, and mechanical work upon post-transplantation reperfusion [8]. The differences between Somah, Celsior®, and Perfadex® are summarized in Table 1. Properties of these three preservation solutions are fairly similar, however, Somah contains many additional protective and unique substrates, such as adenosine, insulin, and abscorbic acid, among others [8]. For these reasons, bioenergetic profiles were generated to further explore mitochondrial physiology and the effects of storage solutions on organ preservation.

**Table 1:** Comparison of organ preservation solutions

	Celsior^a^	Perfadex^a^	Somah^b^
IC/EX	EX	EX	EX
Na^+^	100	138	125
K^+^	15	6	7
Impermeant/Colloid	LactoB, mannitol	Dextran	LactoB, mannitol
Buffer	Histidine	Phos	Bicarb
Antioxidant	GSH, mannitol	-	GSH, mannitol
Osmolarity (mOsm/L)	320	292	305
Ca^2+^	0.25		0.25
Mg^2+^	13	0.8	13
CI^-^	-	142	-
Glucose	-	5	11
Others		SO_4_^2-^0.8 dextran 40 g/L	2 mmol/L adenosine insulin 10 mg/ml 1 mmol abscorbic acid

All units expressed in mmol/L unless otherwise indicated

Source ^a^Reference [8]; ^b^Reference [5]

*Abbreviations*: *IC* intracellular, *EX* extracellular, *Und* undetermined, *LactoB* lactobionate, *HES* hydroxyethyl starch, *Phos* phosphate, *Bicarb* bicarbonate, *GSH* glutathione

In the present study, we sought to compare the biochemical differences between organ preservation solutions and their effectiveness on preserving cellular metabolic and mitochondrial function. Our protocol involves putting cells in suspended animation for 4 to 8 h at 4^o^ or 21 °C, reanimating the cells over 1 h at 37 °C with regular media, and then immediately recording the bioenergetics thereafter. This study evaluates mitochondrial function under clinically relatable conditions, investigates mitochondrial safeguarding, and examines the bioenergetic state of cells after exposure to Celsior®, Perfadex®, and Somah storage solutions.

## Methods

### Microplate coating

Cell-Tak was purchased from Corning (Product #354240). Cell-Tak was prepared in a 2:1 ratio to 1 M NaOH. The wells of a 96-well Seahorse microplate (Seahorse Bioscience, North Billerica, MA) were coated with 40 μl Cell-Tak solution and the plate was allowed to dry at room temperature for 20 min. Following the drying period, wells were aspirated and washed twice with 1x DMEM. The coated microplates were stored at 4 °C and used within 72 h.

### Preservation solution treatments

Rat embryonic myoblasts, H9C2s (ATCC® CRL­1446™), were maintained at standard cell culture conditions in DMEM medium enriched with 10% FBS (Seradigm, Product # 1400-500), 5% L-glutamine (Corning, Lot # 25005289), and 1% Antibiotic-Antimicotic Solution (Sigma Aldrich, catalog # A5955).

H9C2s were seeded in the previously described Cell-Tak coated 96-well Seahorse microplates at 25,000 cells/well. The seeded microplates were incubated for 48 h at 37 °C before introducing experimental conditions to the cells. Each well was washed with 1X PBS (phosphate buffered saline) prior to the addition of 200 μl of experimental solution or control media. Experimental groups included Celsior® (Sanofi, Bridgewater, NJ), Perfadex® (XVIVO Scientific Animation, Wethersfield, CT), Somah (Somahlution®, Jupiter, FL) solutions, 1X PBS, and standard H9C2 media. The microplates were then exposed to either 4 °C or 21 °C for 4-h and 8-h time points. The 10 experimental groups were named 4CEL, 21CEL, 4PER, 21PER, 4SOM, 21SOM, 4PBS, 21PBS, 4MED and 21MED to indicate the temperature condition and storage solution of each group: CEL = Celsior®, PER = Perfadex®, SOM = Somah, and MED = media. Each treatment group consisted of 12 wells and each microplate included all of the experimental groups. Each experimental condition was repeated twice using freshly made solutions and a separately started H9C2 cell line. After each experiment, supernatant from each group was collected and snap-frozen for High Performance Liquid Chromatography (HPLC) analysis.

### Mitochondrial stress test

Immediately after administering the experimental treatments, mitochondrial respiration of H9C2 cells was assessed using XFe96 Extracellular Flux Analyzer (Seahorse Biosciences, North Billerica, MA). Oxygen consumption rates (OCR) were measured in the presence of oxidative phosphorylation (OXPHOS) driving substrates. After 3 basal measurements, 3 measurements each were taken after the addition of oligomycin, FCCP, and rotenone/antimycin A combination. These injected drugs block ATP synthase, uncouple the oxygen consumption from ATP synthesis, and block mitochondrial complexes I and III, respectively. Results were then used to calculate the respiratory control ratio (RCR) and coupling efficiency (CE), which were the primary outcome measurements along with basal respiration. RCR was calculated by the following equation: proton leak ÷ maximal respiration; and CE by the following equation: [(basal respiration – proton leak) ÷ (basal respiration)] * 100.

### Statistical analysis

The data were analyzed as a split-plot design. A plate was considered a “whole plot” with treatments: time, temperature, and the time-temperature interaction. Each solution was considered the split-plot treatment with individual wells used as the experimental unit for a solution. Where there was a statistically significant (alpha = 0.05) effect of the solution, Tukey’s HSD was used to compare all pairs of solutions for differences in mean response. Where there was a statistically significant interaction effect involving a solution, a slice was conducted to test the significance of drug at each level of time, temperature, and/or time-temperature combination and all pairs of solutions were tested for differences in mean response at each level of the time and/or temperature, again, using Tukey’s HSD multiple comparison procedure. Initial analysis suggested non-constant variance among treatment combinations. Therefore, a weighted analysis was conducted using the inverse variance for each solution treatment-plate combination as the weight. All analyses were conducted using SAS PROC MIXED.

## Results

**Fig. 1:**
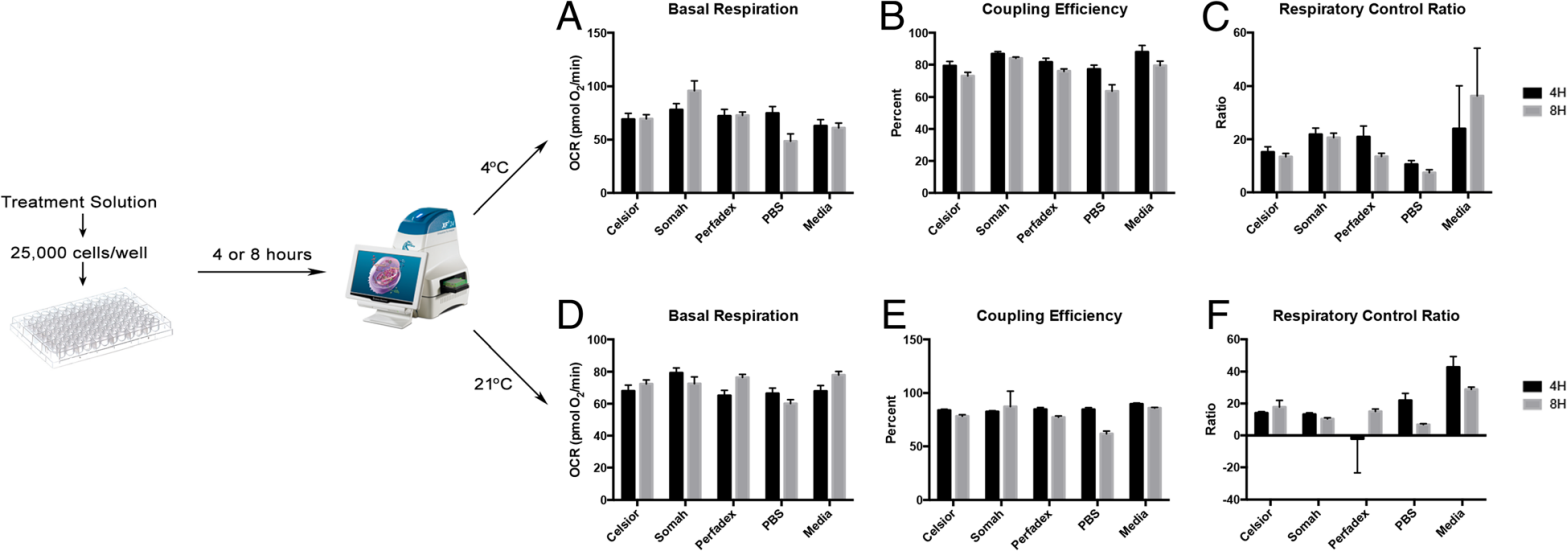
Experimental Design Flow Chart. The conditions of the experiment were performed as shown, with data plotted for both 4 and 8 h time points under 4 °C in panels **a**-**c** and under 8 °C in panels **d**-**f**

**Fig. 2:**
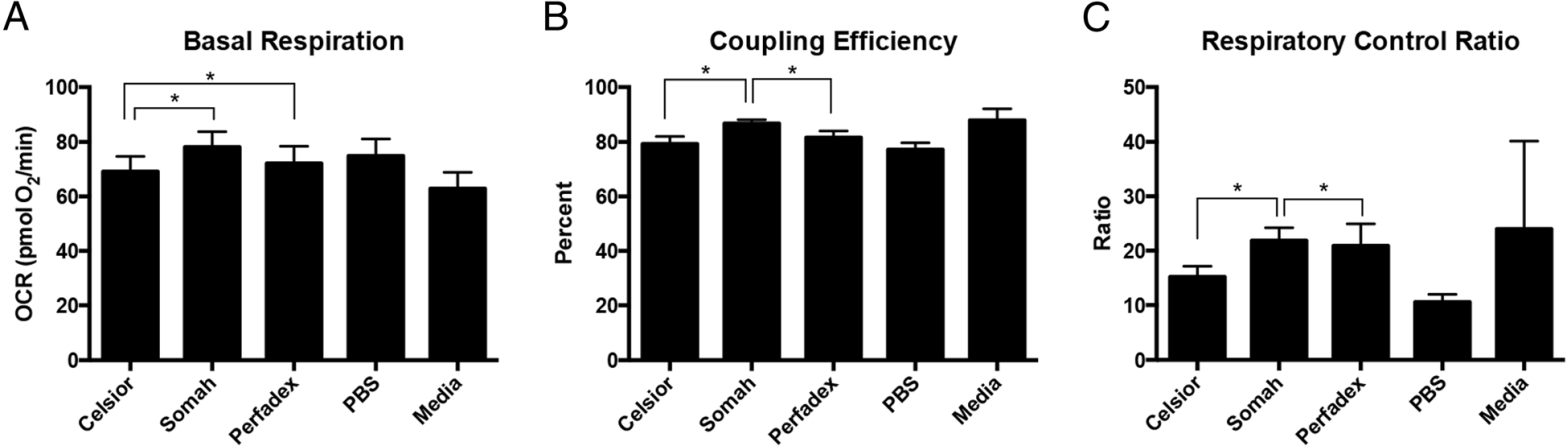
Experimental Conditions at 4 h in 4 °C. For basal respiration (**a**), coupling efficiency (**b**) and respiratory control ratio (**c**), there is significance between the Somah-Celsior® pairwise comparisons. For basal respiration (**a**), there is significance between Celsior- Perfadex pairwise comparisons ®. For coupling efficiency (**b**) and respiratory control ratio (**c**), there is significance between Somah-Perfadex® pairwise comparisons. Error bars shown are SEM

**Fig. 3:**
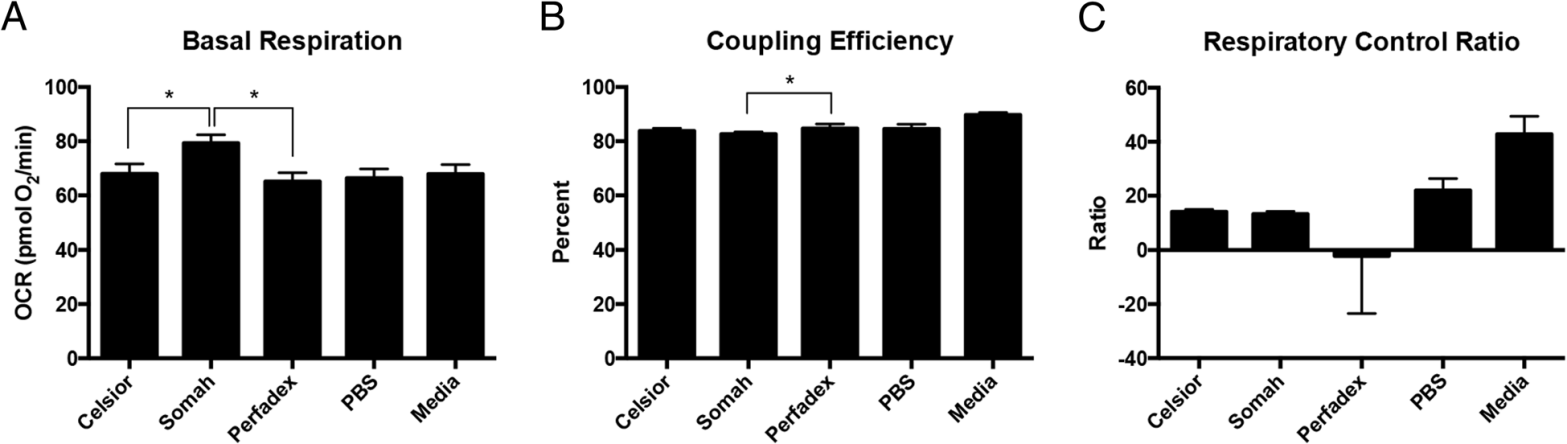
Experimental Conditions at 4 h in 21 °C. For basal respiration (**a**), there is significance between the Somah-Celsior® and Somah-Perfadex® pairwise comparisons. For coupling efficiency (**b**), there is significance between the Somah-Perfadex® pairwise comparison. For respiratory control ratio (**c**), there is no significance between the pairwise comparison of the organ preservation solutions (Somah, Celsior® and Perfadex®). Error bars shown are SEM

**Fig. 4:**
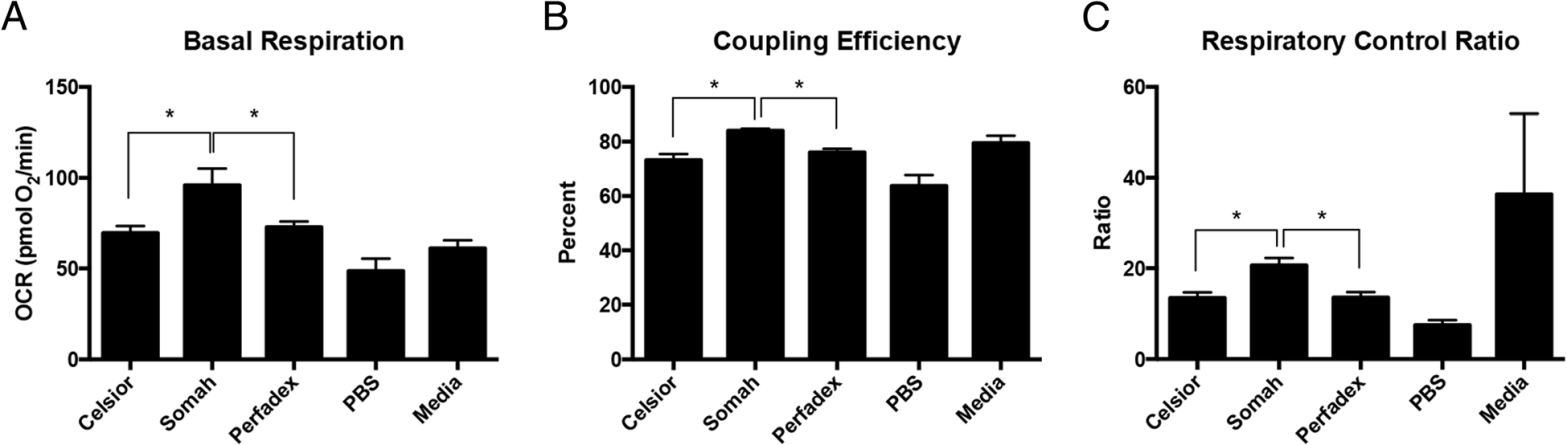
Experimental Conditions at 8 h in 4 °C. For basal respiration (**a**) coupling efficiency (**b**) and respiratory control ratio (**c**), there is significance between the Somah-Celsior®, and Somah-Perfadex® pairwise comparisons. Error bars shown are SEM

**Fig. 5:**
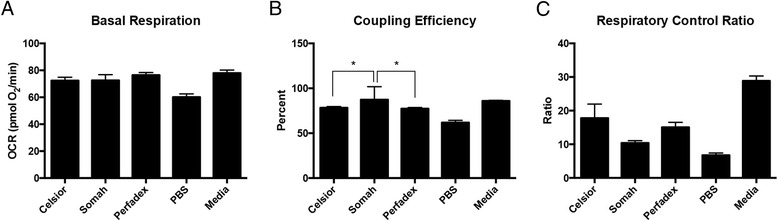
Experimental Conditions at 8 h in 21 °C. For basal respiration (**a**) and respiratory control ratio (**c**), there is no significance between the pairwise comparisons of the organ preservation solutions. For coupling efficiency (**b**), there is significance between the Somah-Celsior®, and Somah-Perfadex® pairwise comparisons. Error bars shown are SEM

An overall flow chart of the experimental setup and resultant data plotted under the 4 °C and 21 °C temperature conditions is shown in Fig. 1. Tukey’s HSD was used for pairwise comparisons of solutions for differences in mean response under each respective time and temperature condition. Only organ preservation solution pairwise comparisons with at least a significance of alpha = 0.05 are mentioned in the figure legends for each experimental condition (Figs. 2, 3, 4 and 5).

### Hour experiments

Comparisons of different solutions are presented in Figs. 2 and 3, showing experiments conducted at 4C and 21C, respectively. H9C2 cells in 4SOM and 4PER both showed a higher mean basal oxygen consumption rate (OCR) than cells treated with either 4CEL, without significant differences between 4SOM and 4PER. H9C2 cells in 21SOM solution showed a higher mean basal OCR than H9C2s in 21CEL or 21PER.

At the 4 h time point, 4SOM and 21SOM treated cells had a higher OCR than cells in 4MED and 21MED, respectively. 4SOM also had a higher coupling efficiency (CE) than 4CEL and 4PER, and 21SOM showed a higher CE than 21PER at 4 h. 4SOM showed a higher respiratory control rate (RCR) than both 4CEL and 4PER.

21MED showed decreased CE in comparison to 21SOM. 21MED had higher RCR values than all other solutions.

### Hour experiments

Comparisons of different solutions are presented in Figs. 4 and 5, showing experiments conducted at 4C and 21C respectively. After 8 h, H9C2s in 4SOM had a higher basal OCR than cells in 4PER and 4CEL. The significant elevation in basal OCR seen in 21SOM during the 4 h experiment did not persist after 8 h.

4SOM had a higher CE than 4CEL; 21SOM had a higher CE than 21CEL and 21PER. 4SOM also showed this same RCR trend above 4CEL and 4PER. As seen in the 4 h experiments, 21MED had a lower CE than 21SOM. Finally, 21MED had a higher RCR than the other solutions, in accordance with the trend seen during 4 h experiments.

## Discussion

The goal of this study was to evaluate H9C2 cellular bioenergetics following a protocol designed to simulate cardiac transplantation by using clinically relatable temperatures and time points. To create this scenario, cells were submerged in Somah, Celsior®, or Perfadex® preservation solutions for 4 and 8 h either at room temperature (21 °C) or “on ice” (4 °C). Normal saline (phosphate buffered saline; PBS) without substrates was chosen as the negative control since the osmotic gradient of cells was expected to be preserved with cellular function steadily declining as endogenous energy stores were depleted. In contrast, normal H9C2 media acted as the positive control since this media is supplemented with the ideal growth substrates for this cell line at 37 °C.

While cultured primary cardiomyocytes are a valuable tool for studying the metabolic capacity of the heart, isolated cardiomyocytes can be fragile and difficult to maintain, especially under stress. As a result, the H9C2 cell line was chosen because these cells express multiple CYP genes comparable to the levels found in the human heart [9]. The role of these endogenous CYP metabolites has been shown to be important in the maintenance of cardiovascular health, and therefore offers an unique model for studying the metabolic activity of the heart [9].

It was hypothesized that the ideal preservation solution could create a state of suspended animation, in which cellular function and metabolism could be preserved for an extended period of time with the least amount of resultant metabolic dysfunction. Altered cellular metabolism is a more sensitive indicator of stress than some traditional outcome measures that may take time to accumulate and detect (e.g., DNA and protein analyses). The role of mitochondria in ischemic reperfusion injury during cardiac transplant is becoming increasingly evident [10]. Safeguarding mitochondrial bioenergetics could play a key role in improving transplant outcomes. Thus, to examine this endpoint, extracellular flux analyses were used to compare mitochondrial respiration of H9C2s after each treatment condition. Targeting various complexes and pathways in the mitochondria allowed us to identify any changes in substrate metabolism and mitochondrial (dys)function. Basal respiration was analyzed as a baseline bioenergetic measurement for each experimental condition. The basal respiration is often controlled by high ATP turnover, and in part dictated by substrate oxidation and proton leak [11]. This measure is therefore altered in response to ATP demand. Coupling efficiency and respiratory control ratios were analyzed since both parameters are ratios, and are therefore useful internal controls that are unaffected by differences in cell number. Additionally, changes in CE and RCR are good indicators of cellular and mitochondrial dysfunction.

Overall, Somah had a greater OCR than both treatment and control groups. This finding was statistically significant after 4 h at room temperature (21 °C) compared to Celsior® and Perfadex®. Compared to the control media, the OCR of Somah-treated H9C2s (4SOM and 21SOM) were increased compared to the control media (Figs. 2 and 3) at both 4^o^ and 21 °C after 4 h of incubation. By 8 h, the media and the preservation solution conditions become similar and the significant trend diminishes. 4SOM had an increased OCR value compared to 4PER and 4CEL experimental groups, as well as 4MED and 4PBS controls. Also at the 8-h time point, 21PBS had a significantly lower OCR than the other treatment groups, while 21SOM, 21CEL and 21PBS have similar OCR values. As expected, PBS was overall the least-ideal solution since it has the lowest OCR. At 4 h under both temperature conditions, the differences between all solutions, and notably PBS, were not obvious. This is likely due to the resiliency of the H9C2 cell line, reiterating why this cell line is a good model for investigating metabolic activity and drug metabolism in the heart [9]. An aim for a future study may include longer time points such as 12 or 24 h, though organs stored for extended periods of time are not considered clinically viable.

The basal respiratory OCR of Somah-treated cells was greatest and allowed cells to maintain metabolism at a more consistent OCR through the 4-h time point (Figs. 2 and 4). In comparison, base media, Celsior®, and Perfadex® took up to 8 h to return to a higher OCR at 21 °C (Figs. 3 and 5). These same differences are even more pronounced at 4 °C, where 4CEL is found to have a lower OCR than both 4PER and 4SOM.

Coupling efficiency (CE) is defined as the proportion of mitochondrial respiratory rate used to drive ATP synthesis (e.g., perfectly coupled OXPHOS has a coupling efficiency of 100%) [12]. CE is calculated as the change in basal respiration rate with the addition of oligomycin, and is thus presented as the fraction of basal mitochondrial oxygen consumption used for ATP synthesis. Since it takes the basal respiration into account, the CE also varies with ATP demand and is most sensitive to changes in proton conductance. Since CE is a ratio of two rates, it is an internally normalized value and can indicate mitochondrial dysfunction. Overall, both Somah and media treated cells have a high CE (90%), which shows that even though maximal respiration may be damaged (see RCR), the normal ATP turnover when compared to the leak of protons over the membrane has not changed. After both 4 and 8 h at 4 °C, the CE of H9C2s treated with either Somah or media was significantly higher than all other solutions (Figs. 2 and 4) and Celsior® and Perfadex® were not significantly different at either time point. The shorter time point of 4 h led to a higher CE in Somah over the other solutions. 4SOM is comparable to the media control condition, while at 21SOM the CE was significantly lower than compared to Celsior® and Perfadex®, yet the CE of media treated cells remained significantly higher than the other groups. This might be due to an increased proton leak or a decrease in ATP production. If caused by a rise in proton leak, there might be an increased production of superoxide anions and other ROS that cause tissue or organ damage. In previous research, Somah has shown to function best at 21 °C [8]. This is an interesting link to our significantly lowered CE result and should be the subject of future research. However, given the similarities between Somah and media treatments in the hypothermic conditions of this experiment, Somah is considered preferred at 4 °C, while media is favored at a warmer temperature.

The respiratory control ratio (RCR) is calculated as a ratio of the uncoupled rate of mitochondrial respiration to the rate with ATP synthase inhibited by oligomycin. The RCR is sensitive to changes in substrate oxidation and proton leak, which makes it sensitive overall to potential dysfunction [11]. One major benefit of the RCR as a measurement is that it is internally normalized, similar to CE. At 4 h, 4SOM results in a higher RCR than H9C2s treated with either 4CEL or 4PER, but has a similar RCR to media. Similar to the CE results, this change is attributable to either an increase in proton leak or a decrease in maximal ATP turnover, or a combination of both. Unlike the CE results, the RCR of Somah-treated cells remains significantly higher than all of the other solutions over the entire 8 h. 21MED also has a high RCR over both 4 and 8 h, with significance over all other treatment groups. As expected by 8 h, 21PBS has the lowest RCR of all groups. The experimental group most similar to the media-treated cells was the Somah-treated cells. While Somah best maintained a high RCR under hypothermic conditions, media maintained a high RCR at warmer room temperature conditions.

A notable limitation of the study is the limited number of storage solutions included in the comparison. As previously mentioned, there are numerous organ storage solutions currently available, and even more in development. Therefore, an aim in future studies includes incorporating more organ storage solutions into this experimental design, including UW solution, Custodiol, histidine tryptophan ketogluterate (HTK), and Collins storage solutions.

Another limitation was the use of non-human tissue for the measurement of bioenergetic values. While cardiomyocytes of multiple animal models closely resemble that of a human, it can still be difficult to draw solid conclusions regarding the clinical value of the findings presented. Even though this concern is valid, this study demonstrates a reliable and elegant method of measuring the bioenergetic profile of cardiac cells lines during storage in various solutions. Therefore, based on the methodology and experimental results, the results from this experiment can be extrapolated for translation to human tissue. It is important to establish non-human models since healthy human cardiac tissue is scarcely available. Despite these limitations as a clinical model, the results of this study suggest differences in metabolic preservation between the solutions, with Somah being the optimal preservation solution.

Additionally, the model explored in this study is limited by the fact that the cells are seeded in a monolayer, and not multi-layered as seen in an organ. The use of tissue biopsies has been very challenging, as the mitochondrial stress test performed in these assays is an extremely precise procedure, in which exact cell number are crucial when injecting different drugs in the wells of the assay plate.

Future studies to use multi-layer cardiac myoblast culture as well as creating micro-tissues by co-culture with fibroblasts and cardiac myocytes would provide key three-dimensional bioenergetic data. Currently, these culture techniques are being developed with collaborators, as extracellular flux analyzers currently do not provide consistent and homogenous data that is reportable for these types of cultures. The benefit, on the other hand, of a single layered model is that these assays provide very accurate results, without any interference of other processes. Thus, these monolayer experiments serve as the first confirmatory step that protecting cellular mitochondrial integrity of transplantable organs may lead to improved organ function, reduced IRI and better transplant outcomes.

## Conclusions

These results suggest Somah solution sustains a higher and more consistent OCR when compared to both Celsior® and Perfadex® at 4 °C, and these effects can last from 4 h up to 8 h. These findings are additionally supported by current literature [8]. Given these results, Somah offers benefits over the solutions at 4 °C and should be a target in future organ preservation solution research.

## Additional file

Additional file 1: The raw data for each experimental condition used for statistical analysis is summarized in this data set. Conditions include incubation of cardiac cells in control and preservation media at 4 degrees C or 21 degrees C for 4 hours or 8 hours. Control groups were incubated in either media or phosphate-buffered saline (PBS), and treatment groups were incubated in either Celsior, Somah, or Perfadex. (XLSX 80 kb)

## Abbreviations

CE: Coupling efficiency
CEL: Celsior
HTK: Histidine tryptophan ketogluterate
IRI: Ischemic reperfusion injury
MED: Media
OCR: Oxygen consumption rate
PBS: Phosphate buffered saline
PER: Perfadex®
RCR: Respiratory control rate
ROS: Reactive oxygen species
SOM: Somah
UW: University of Wisconsin solution
